# Retinal microvascular phenotypes can track small vessel disease burden and CPAP treatment effectiveness in obstructive sleep apnoea

**DOI:** 10.1177/0271678X241291958

**Published:** 2024-11-02

**Authors:** Ylenia Giarratano, Elizabeth A Hill, Charlene Hamid, Stewart Wiseman, Calum Gray, Francesca M Chappell, Roberto Duarte Coello, Maria C Valdés-Hernández, Lucia Ballerini, Michael S Stringer, Michael J Thrippleton, Daniela Jaime Garcia, Xiaodi Liu, William Hewins, Yajun Cheng, Sandra E Black, Andrew Lim, Rosa Sommer, Joel Ramirez, Bradley J MacIntosh, Rosalind Brown, Fergus Doubal, Tom MacGillivray, Joanna M Wardlaw, Renata Riha, Miguel O Bernabeu

**Affiliations:** 1 Centre for Medical Informatics, Usher Institute, The University of Edinburgh, Edinburgh, UK; 2 Centre for Clinical Brain Sciences, Edinburgh Imaging, UK Dementia Research Institute Centre, The University of Edinburgh, Edinburgh, UK; 3 School of Applied Sciences, University of the West of England (UWE), Bristol, UK; 4 Department of Humanities and Social Sciences, University for Foreigners of Perugia, Perugia, Italy; 5 Division of Neurology, Department of Medicine, The University of Hong Kong, Hong Kong SAR, China; 6 Department of Neurology, West China Hospital, Sichuan University, Chengdu, China; 7 Sunnybrook Health Sciences Centre, Toronto, Canada; 8 Sleep Research Unit, Department of Sleep Medicine, Royal Infirmary of Edinburgh, Edinburgh, UK; 9 The Bayes Centre, The University of Edinburgh, Edinburgh, UK

**Keywords:** Retinal imaging, OCT-angiography, OSA, SVD burden, CPAP treatment

## Abstract

Optical coherence tomography angiography (OCT-A) retinal imaging enables *in vivo* visualization of the retinal microvasculature that is developmentally related to the brain and can offer insight on cerebrovascular health. We investigated retinal phenotypes and neuroimaging markers of small vessel disease (SVD) in individuals with obstructive sleep apnoea (OSA). We enrolled 44 participants (mean age 50.1 ± SD 9.1 years) and performed OCT-A imaging before and after continuous positive airway pressure (CPAP) therapy. Pre-treatment analyses using a generalized estimating equations model adjusted for relevant covariates, revealed perivascular spaces (PVS) volume in basal ganglia associated with greater foveal vessel density (fVD) (p-value < 0.001), and smaller foveal avascular zone area (p-value = 0.01), whereas PVS count in centrum semiovale associated with lower retinal vessel radius (p-value = 0.02) and higher vessel tortuosity (p-value = 0.01). A reduction in retinal vessel radius was also observed with increased OSA severity (p-value = 0.05). Post-treatment analyses showed greater CPAP usage was associated with a decrease in fVD (p-value = 0.02), and increased retinal vessel radius (p-value = 0.01). The findings demonstrate for the first time the potential use of OCT-A to monitor CPAP treatment and its possible impact on both retinal and brain vascular health.

## Introduction

Obstructive sleep apnoea (OSA) is a significant healthcare burden that affects 1.5 million adults in the UK and 935 million worldwide.^1^ It is characterized by repeated interruptions of normal breathing caused by complete (apnoea) or partial (hypopnoea) collapse of the upper airway during sleep, resulting in intermittent hypoxia and sleep fragmentation. Intermittent hypoxia during sleep can lead to inflammatory processes, oxidative stress, and decline in brain health. As a result, individuals with OSA may be at increased risk of developing cerebral small vessel disease (SVD).^2,3^

Optical coherence tomography angiography (OCT-A) is a fast and efficient retinal imaging technique that enables the visualization of the retinal microvasculature near the brain. The homology and proximity of cerebral and retinal microvasculature have led to significant interest in exploring whether quantifiable changes in the retinal vessels are indicative of alterations in cerebrovascular health.^4,5^ Indeed, increasing evidence suggests that retinal changes can be observed in patients with brain disorders like Alzheimer's disease (AD),^6^ Parkinson's disease (PD),^7^ multiple sclerosis (MS),^8^ and stroke.^9^ In studies on SVD, researchers have reported abnormalities in retinal fundus photography, such as arteriovenous nicking, retinal bleeding, and narrowed arteries, which were linked to increased white matter hyperintensities (WMH) and enlarged perivascular spaces (PVS), markers of SVD burden.^10,11^ However, there is still limited research on OCT-A retinal changes in SVD.^12^ Therefore, in this study we carried out an investigation of retinal phenotypes, captured with this advanced imaging modality, and brain markers of SVD in individuals with OSA.

Previous retinal investigations using fundus photography in individuals with OSA have reported a progressive arterial narrowing, increased vascular tortuosity, and reduction in fractal dimension associated with OSA severity.^13–17^ Using optical coherence tomography (OCT), an imaging modality that provides cross-sectional images of the retinal layers, studies have observed a decrease in peripapillary retinal nerve fiber layer (pRNFL) thickness and an increase of the macular thickness in the foveal, nasal, and temporal regions in individuals with OSA when compared to healthy controls.^18–20^

OCT imaging technology has also been utilized to assess changes in thickness of retinal layers after the use of continuous positive airway pressure (CPAP) therapy, the recommended first-line clinical intervention for OSA in adults.^21–23^ Studies have suggested an overall thickening of the pRNFL and of the inner and outer regions of the macula associated with CPAP usage.^24,25^ However, different outcomes have also been reported in response to CPAP treatment depending on the severity of the condition. In patients with mild/moderate OSA, retinal swelling was observed, and after CPAP treatment, a reduction in thickness was reported in the foveal region, inner and outer macular regions, and overall macular volume. On the other hand, individuals with severe OSA showed retinal atrophy, but an increase in foveal macular thickness was observed after CPAP therapy.^26^ It is worth mentioning that, despite many studies not considering CPAP compliance, effectiveness of the intervention relies on patient adherence, and better outcomes are achieved when CPAP is used for at least 4 hours per night.^27^

More recent investigations have involved the use of OCT-A imaging to assess changes in vascular density (VD) in patients with OSA through cross-sectional case-control studies. Despite these efforts, conflicting results have been reported. Notably, in individuals with severe OSA, some investigations have observed a reduction in VD within both the superficial vascular complex (SVC) and deep vascular complex (DVC),^18,28^ whereas other studies have reported an increased VD in either SVC or DVC.^29,30^ The inconsistency in these findings highlights the need for further research on OCT-A vascular changes associated with OSA. Furthermore, to the best of our knowledge, the use of this retinal imaging modality for monitoring CPAP treatment is still unexplored.

In the current study, we undertake a deep phenotyping of retinal microvascular characteristics in patients with moderate and severe OSA using OCT and OCT-A. As retinal microvasculature alterations might mirror cerebral vascular dysfunctions, we investigate associations between retinal phenotypes and neuroimaging markers of SVD. We analyze associations of sleep variables, indicators of OSA severity, and retinal microvascular measurements beyond vascular density and retinal thickness. Finally, considering the gap in the literature on the use of OCT-A to monitor CPAP therapy and the crucial role of treatment adherence for efficacy, we further explore the relationship between OCT-A phenotype longitudinal changes and the amount of CPAP usage.

In this research, we propose the application of OCT-A retinal imaging for the first time as a valuable tool for monitoring effectiveness of treatment for SVD using CPAP as exemplar. This is based on the hypothesis that changes in retinal microvascular phenotypes may be instrumental in assessing retinal vascular health and, by extension, cerebral vascular health.

## Materials and methods

### Participants

Adult participants (aged ≥18 years) were recruited from a tertiary clinical sleep service at the Department of Sleep Medicine, Royal Infirmary of Edinburgh. Eligibility criteria included new clinical diagnosis of OSA, no previous CPAP treatment, ability to undergo MRI, Epworth Sleepiness Score ≥11/24,^31^ and modified Rankin Score <3.^32^ Individuals with neurological or psychiatric conditions, panic disorder, renal impairment, persistent non-sinus arrhythmia, severe pulmonary or cardiac disease, pregnant or breastfeeding, were ineligible. Further exclusions were contraindications to MRI, gadolinium contrast agent, oxygen saturation below 90% upon waking, arterial oxygen saturation <90% on room air, and the use of alpha blocking agents which can interfere with measurement of peripheral arterial tone. Clinical diagnosis of OSA was made by experienced sleep clinicians based on clinical history and objective testing (Level 1 attended polysomnography (PSG) or Level 3 home sleep apnoea testing (HSAT) as per local clinical practice).^33^

The West of Scotland Research Ethics Service approved the study (REC reference 17/WS/0198), and all procedures were conducted according to the Declaration of Helsinki. All participants gave written informed consent.

### Clinical variables

We recorded age, sex, blood pressure, body mass index (BMI), history of diabetes, smoking, hypertension, and hypercholesterolemia status. All participants underwent a single night of HSAT at baseline using the WatchPAT™200 (Itamar Medical Ltd., Cesarea, Israel), a portable device for home OSA diagnosis. This device employs proprietary software that utilizes a physiological signal, known as the peripheral arterial tone (PAT), in conjunction with other physiological measurements, including heart rate and oximetry, to provide comprehensive indices for accurately diagnosing sleep apnea. We reported the PAT-derived apnea-hypopnea index (pAHI) which describes the number of PAT-derived breathing pauses per hour of sleep. This index is clinically used to assess the severity of the condition, categorized as mild (5–14 events/hour), moderate (15–30 events/hour), and severe (>30 events/hour).^22^ We also reported the mean oxygen saturation (SpO2) and the nadir of SpO2. A second single night of HSAT was completed after at least 4 months on CPAP therapy.

CPAP was initiated after the first HSAT recording night. Patient education and mask fitting was completed by an experienced Registered Polysomnographic Technologist (RPSGT). All participants were issued with an auto-titrating CPAP device with built-in humidifier (ResMed Airsense™ 10 AutoSet, ResMed UK Ltd., Didcot, UK), and monitored using ResMed AirView™ telemonitoring software (ResMed AirView version 4.12.0–6.0). CPAP usage was monitored remotely by the RPSGT and patients followed up by phone or email contact to optimize compliance. CPAP adherence was defined as at least 4 hours per night, with optimal adherence identified as patients who used treatment for at least 4 hr/night on 80% of nights.^34^

### Retinal imaging and retinal phenotypes

**Figure 1.**
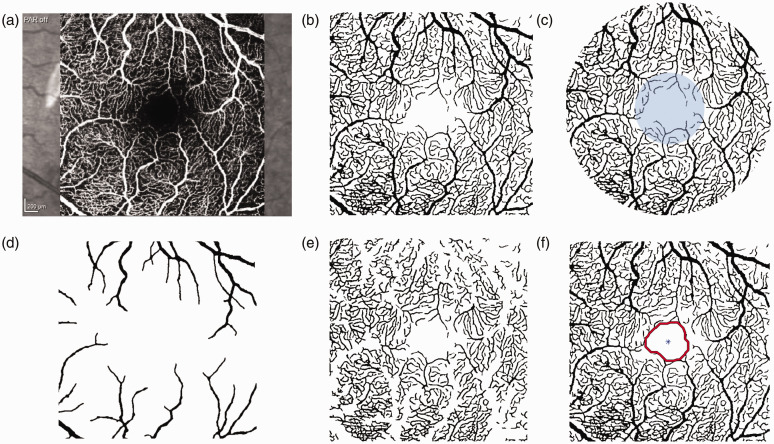
OCT-A processing. (a) OCT-A scan; (b) Binary image; (c) Parafoveal ring and foveal circle (blue); (d) Vessels with caliber greater than 20 µm; (e) Vessels with caliber smaller than 20 µm and (f) FAZ detection.

Retinal imaging was performed using the SPECTRALIS system (Heidelberg Engineering, Germany), an imaging platform able to capture both OCT and OCT-A scans. When possible, images of both eyes were acquired from each participant at each visit. Follow-up imaging was scheduled at the same time of day as the baseline acquisition whenever feasible, and at least 4 months into treatment. OCT scans centered at the optic nerve were used to assess peripapillary RNFL thickness, and volumetric OCT scans across retinal layers were performed to examine macular thickness in the parafoveal regions of interest (Supplementary Figure S1). OCT-A quality score provided by the SPECTRALIS device was recorded for each eye. Images of the SVC centered at the macula with 3 mm × 3 mm field of view (Figure 1(a)) were processed using a segmentation pipeline validated in our previous work.^35^ Briefly, the optimally oriented flux (OOF) filter was used for vessel enhancement, and then thresholding was applied for binarization.^36^ All segmentations and computational measurements underwent visual inspection and edited to remove errors as needed.The vasculature captured in the binary images was modeled as a graph to extract structural and functional metrics (Figure 1(b)). To account for vessels at different scales, large and small vessels, two different networks were generated by selecting a threshold for vessel caliber (20 µm).^37^ *Vessel density* in *parafoveal ring* and in the *foveal circle* were computed as the ratio of the white pixels over the total number of pixels of that region of interest (Figure 1(c)). *Average radius* in *large* and *small vessels* was obtained by computing the Euclidean distance from the central pixel in the vasculature to the closest tissue (black) pixel (Figure 1(d) and (e)). *Foveal avascular zone (FAZ) size* was defined as the largest area free from vessels in the central region of the scan (Figure 1(f)). *Average vessel tortuosity* was obtained by calculating the ratio between the graph length and the Euclidean distance between the start and ending point of each vessel, and *branching points* were found as nodes in the network with degree of three. *Peripapillary RNFL global thickness* and *macular thickness* in the foveal, superior, nasal, inferior, and temporal regions were calculated by the built-in device software. Finally, since OCT-A does not provide flow information directly, surrogate hemodynamics measurements were computed as functional metrics. Considering vessel bifurcations play a significant role in determining the flow characteristics of blood, including the degree of phase separation between plasma and red blood cells, and the distribution of shear stress within the vessels,^38^ we computed the *average bifurcation distance* as the mean vessel length between branching points. Additional functional information from OCT-A images can be obtained using fluid dynamic concepts like Murray’s law, which describes how network branching minimizes energy for fluid transport.^39,40^ In the vascular system, it explains that blood vessel diameters are arranged to reduce the heart's workload while ensuring the supply of nutrients and oxygen to tissues. In healhy retinal vasculature, Murray's law is described by the formula: $\mathrm{Radiu}s_{\mathrm{parent}\;\mathrm{vessel}}^{3}=\mathrm{Radiu}s_{\mathrm{branch}1\;}^{3}+\;\mathrm{Radiu}s_{\mathrm{branch}2}^{3}$ .^41^ Deviations from it can indicate changes in normal branching patterns and vessel diameters, leading to inefficient blood flow and potential retinal damage.^42^ Therefore, we computed the Kolmogorov-Smirnov score (KS) as a measure of the deviation from Murray's law in our vascular networks. This is achieved by calculating the distance between the distribution of the cubes of the radii of parent vessels and the distribution of the sum of the cubes of the radii of the daughter vessels. A higher KS score signifies a greater deviation from the optimal branching structure outlined in Murray's law. Finally, we further characterized the vascular branching pattern by computing the symmetry ratio, an index that describes the similarity between the caliber of two branches after a bifurcation point (branch_2_/branch_1_). This ratio affects the distribution of blood flow, pressure gradients, and overall vascular resistance. While a certain level of symmetry is expected in healthy blood vessels, excessive or abnormal symmetry can impact blood flow patterns and potentially lead to disorders or diseases.^42,43^ All images were processed and analyzed using MATLAB (MathWorks, Natik, MA, USA; version 2020 b), and Python (version 3.10.6, Python Software Foundation, Wilmington, DE, USA).

### Brain imaging and SVD markers

MRI scans were acquired on a 3T Siemens MAGNETOM Prisma scanner at the Edinburgh Imaging Facility Royal Infirmary of Edinburgh, before and after at least 4 months into treatment. Only baseline scans were included in this study. The imaging protocol and processing have been previously published.^44,45^ All visual assessments of SVD on MRI were carried out in accordance with the STRIVE guidelines.^46^ We used a previously validated analysis pipeline to assess intracranial volume (ICV), brain tissue volume, white matter hyperintensities (WMH) volume and the Fazekas scale (periventricular WMH score plus deep WMH score), to visually rate WMH severity.^44,47^ WMH volume was normalized for intracranial volume (ICV) and log-transformed given the highly skewed distribution. We also assessed perivascular spaces (PVS) using computational measures of total volume, count, average individual size, length, and width in the basal ganglia (PVS-BG) and centrum semiovale (PVS-CSO).^48–50^ Boundaries of regions of interest were individually checked and manually rectified if necessary. PVS volume measurements were further corrected for the volume of the corresponding region of interest. Lacunes and brain microbleeds, also markers of SVD, were either absent or present in very few participants. This is typical for a cohort with these characteristics, and as a result, they were not included in our analyses. Finally, superficial and deep brain atrophy visual scores were obtained by comparing the enlargement of the sulci and the ventricles of each participant against a template of normal reference brains.^51^ All computational measures were visually inspected and edited to remove errors as necessary.

### Statistical methods

Associations at baseline between retinal measurements and SVD markers were investigated using the generalized estimating equations (GEE) approach to account for both eyes in one analysis without violating any statistical modelling assumptions. Age, sex, systolic blood pressure, BMI, self-reported diabetes status, hypercholesterolemia, smoking status, and retinal image quality index were included as covariates. Associations at baseline between retinal phenotypes and sleep variables were similarly analyzed. Standardized coefficients were used to compare the strength of associations without concerning about the scales of the raw data.

Two-tailed *t-test* and *Mann-Whitney U* test, independently for each eye, were used to investigate longitudinal retinal changes between patients with optimal (mean usage ≥4 hr/night) and sub-optimal (<4 hr/night) CPAP adherence. Additionally, longitudinal analyses adjusted for covariates were carried out using the amount of change in retinal phenotypes (difference in value between visits) as dependent variable and CPAP usage (average hours per day) as the independent variable in the GEE model.

Finally, a further analysis was carried out using macular thickness measurements obtained from the OCT device. Considering the pAHI an indication of OSA severity, we categorized participants as either moderate or severe cases. We then compared macular thickness in different regions of interest between the two groups cross-sectionally using the two statistical tests previously described. Focusing on the severe OSA group, which has the largest sample size, longitudinal thickness changes were analyzed using a paired *t-test* in patients with optimal and sub-optimal adherence. Considering the exploratory nature of this work, p-values are reported as uncorrected for multiple comparisons. Missing data were excluded from the relevant analyses. All statistical analyses were performed in R Studio using R version 3.6.3.

## Results

### Patient demographics

Among 44 participants enrolled, two participants withdrew from the study, one before the collection of data and another after the first assessment. A total of 42 participants with brain MRI completed the OCT assessment and 40 underwent OCT-A retinal assessment before initiating CPAP treatment. Thirty-seven participants contributed with OCT-A scans of both eyes.

Patient demographic data and brain imaging measurements at baseline are summarized in Table 1. The mean age was 50.1 ± (SD) 9.1 years. There were 14/43 (33%) females, 14/43 (32.6%) smokers, 4/43 (9%) reported diabetes, 17/43 (40%) reported hypertension, and 12/43 (28%) reported hypercholesterolemia. Mean pAHI value was 53.7 ± (SD) 24.7, including 33/43 severe and 10/43 moderate cases. Median WMH volume was 0.99 ml (interquartile range IQR: 0.58–1.73) and median Fazekas scale was 2 (IQR: 1–2).

**Table 1.** Sleep apnoea study demographics at baseline.

		Participants (N = 43)	
Age, years, mean (SD)		50.1 (9.1)	
Female, n (%)		14 (33%)	
Hypertension, n (%)		17 (40%)	
Hypercholesterolemia, n (%)		12 (28%)	
Diabetes, n (%)		4 (9%)	
BMI (kg/m^2^), mean (SD)		34.8 ± 6.1	
Smoker status: current v. ex v. never, n (%)		8 (18.6%) v. 14 (32.6) v. 21 (48.8%)	
pAHI, events/hr, mean (SD)		53.7 (24.7)	
SpO_2_ mean, mean (SD)		92.9 (2.0)	
SpO_2_ nadir, mean (SD)		75.7 (9.3)	
Neuroimaging		N = 42	
Intracranial volume (ml), mean (SD)		1476.04 ± 161.37	
WMH volume (ml), median (IQR)		0.99 (0.58–1.73)	
Fazekas scale (0:6), median (IQR)		2 (1–2)	
Brain volume (ml), mean (SD)		1140.32 ± 118.14	
Superficial atrophy (0:6), median (IQR)		1 (1–2)	
Deep atrophy (0:6), median (IQR)		1 (1–2)	
Number of lacunes, median (IQR)		0 (0–0)	
Number of microbleeds, median (IQR)		0 (0–0)	
PVS volume in BG (%ROIV), median (IQR)		1.95 (1.60–2.55)	
PVS volume in CSO (%ROIV), median (IQR)		3.00 (2.08.−4.10)	
PVS count in BG, median (IQR)		122 (104–167)	
PVS count in CSO, median (IQR)		608 (456–730)	
PVS size in BG (ml), median (IQR)		0.02 (0.01–0.02)	
PVS size in CSO (ml), median (IQR)		0.02 (0.01–0.02)	
PVS length in BG (voxel), mean (SD)		6.38 ± 0.44	
PVS length in CSO (voxel), mean (SD)		6.39 ± 0.57	
PVS width in BG (voxel), mean (SD)		3.03 ± 0.27	
PVS width in CSO (voxel), mean (SD)		5.70 ± 0.62	
	CPAP treatment (n = 42)		
CPAP usage, hr/night, mean (SD)	5.1 ± 3.0		
Total days of treatment, mean (SD)	135.4 ± 40.2		
	Sub-optimal CPAP use (n = 23)	Optimal CPAP use (n = 19)	p-value
Severe OSA, n (%)	16 (70%)	16 (84%)	0.46
CPAP usage, hr/night, mean (SD)	3.7 ± 3.4	6.7 ± 0.9	<0.01
Total days of treatment, mean (SD)	142.5 ± 51.9	127.0 ± 16.2	0.18

BMI: body mass index; pAHI: PAT-derived apnoea-hypopnoea index; SpO_2_: oxygen saturation; WMH: white matter hyperintensity; PVS: perivascular spaces; BG: basal ganglia; CSO: centrum semiovale; IQR: interquartile range. Non-normal distributions are reported as medians (interquartile range, Q1–Q3). CPAP treatment: optimal adherence was defined as CPAP usage at least 4 hr/night for 80% of nights. P-values were computed using the *t-test* for variables with a normal distribution and the *Mann-Whitney U test* for non-normally distributed numerical variables. *Chi-squared test* was used for categorical variables.

Forty-two participants received CPAP treatment and 40 underwent OCT imaging. Out of the initial 40 individuals with OCT-A scans at baseline, follow up images of the right (OD) eye were acquired in 37 participants; whereas 31 individuals contributed with follow up images of the left eye (Supplementary Figure S2). The average duration of treatment was 135.4 ± (SD) 40.2 days. Nineteen individuals used CPAP machine for at least 4 hours per night for 80% of the treatment period. Further details can be found in Table 1 and Supplementary Tables S1–2.

#### Retinal phenotypes are associated with SVD burden

**Figure 2.**
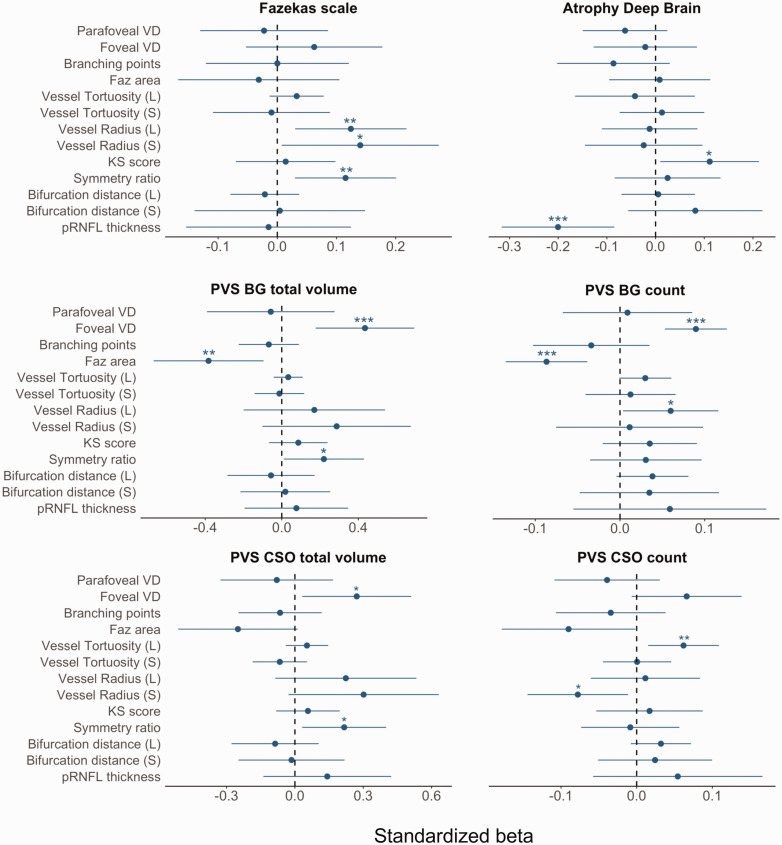
Standardized betas with 95% confidence interval (CI) of retinal and brain variables using GEE model adjusted for age, sex, systolic blood pressure, self-reported diabetes status, BMI, hypercholesterolemia, smoking status, and image quality. PVS volumes were corrected for the volume of the relevant region of interest. PVS: perivascular spaces; CSO: centrum semiovale; BG: basal ganglia. CI containing the value zero (dashed line) indicates an unadjusted p-value > 0.05, while *: p-value ≤ 0.05, **: p-value ≤ 0.01, ***: p-value ≤ 0.001. VD: vessel density, FAZ: foveal avascular zone, KS: Kolmogorov-Smirnov, L: large vasculature, S: small vessels.

Figure 2 shows the standardized coefficients of retinal and SVD markers obtained using the GEE model. Greater Fazekas scale was associated with increased vessel calibers, i.e., large and small vessel radius (β_s_ = 0.12, CI: [0.03, 0.22], p-value = 0.01, and β_s_ = 0.14, CI: [0.13, 0.27], p-value = 0.04), and higher branching symmetry ratio (β_s_ = 0.12, CI: [0.03, 0.20], p-value = 0.01). Increased deep brain atrophy was associated with higher KS score (β_s_ = 0.11, CI: [0.01, 0.21], p-value = 0.03) and lower thickness of the pRNFL (β_s_ = −0.20, CI: [−0.32, −0.08], p-value < 0.001). Increased PVS-BG volume was associated with higher foveal vessel density (β_s_ = 0.43, CI: [0.18, 0.69], p-value <0.001), greater branching symmetry ratio (β_s_ = 0.22, CI: [0.01, 0.44], p-value = 0.04), and lower FAZ area (β_s_ = −0.38, CI: [−0.67, −0.10], p-value = 0.01). Greater PVS-BG count was associated with higher foveal vessel density (β_s_ = 0.09, CI: [0.05, 0.13], p-value < 0.001), greater tortuosity of large vessels (β_s_ = 0.06, CI: [0.01, 0.12], p-value = 0.04), and lower FAZ area (β_s_ = −0.09, CI: [−0.13, −0.04], p-value <0.001). Greater PVS-CSO volume was associated with higher foveal vessel density (β_s_ = 0.27, CI: [0.03, 0.51], p-value = 0.03), and increased branching symmetry ratio (β_s_ = 0.22, CI: [0.03, 0.40], p-value = 0.02). Increased PVS-CSO count was associated with decreased small vessel radius in (β_s_ = −0.08, CI: [−0.14, −0.01], p-value = 0.02) and increased large vessel tortuosity (β_s_ = 0.06, CI: [0.02, 0.11], p-value =0.01). Further associations of measurements characterizing PVS shape can be found in Supplementary Table S3 and Figure S3.

#### Retinal phenotypes are associated with sleep apnoea severity

**Figure 3.**
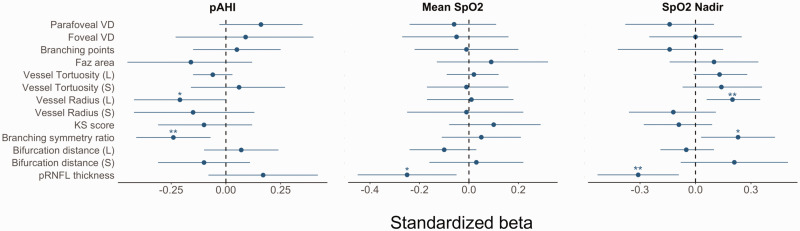
Standardized coefficients with 95% confidence interval (CI) obtained using the GEE model adjusted for age, sex, SBP, self-reported diabetes status, BMI, hypercholesterolemia, smoking status, and image quality. CI containing the value zero (dashed line) indicates a p-value greater than 0.05. *: p-value ≤0.05, **: p-value ≤ 0.01, ***: p-value ≤0.001. pAHI: WatchPAT derived apnoea-hypopnoea index, SpO2: oxygen saturation; VD: vessel density; FAZ: foveal avascular zone; KS: Kolmogorov-Smirnov; L: large vasculature; S: small vessels.

At baseline, cross-sectional analyses showed a relationship between higher pAHI with both lower radius of large vessels (β_s_ = −0.21, CI: [−0.42, 0.00] p-value = 0.05), and lower branching symmetry ratio (β_s_ = −0.24, CI: [−0.41, −0.07], p-value = 0.01). Whereas a positive association was observed between the same retinal features and SpO_2_ nadir (β_s_ = 0.20, CI: [0.06, 0.35], p-value = 0.01, and β_s_ = 0.23, CI: [0.03, 0.43], p-value = 0.03). Lower pRNFL thickness was associated with both higher mean SpO_2_ (β_s_ = −0.25, CI: [−0.45, −0.05], p-value = 0.02) and higher nadir SpO_2_ (β_s_ = −0.31, CI: [−0.53, −0.09], p-value < 0.01) (see Figure 3 and Supplementary Table S4).

#### Longitudinal CPAP adherence predicts amount of change in retinal phenotypes

**Figure 4.**
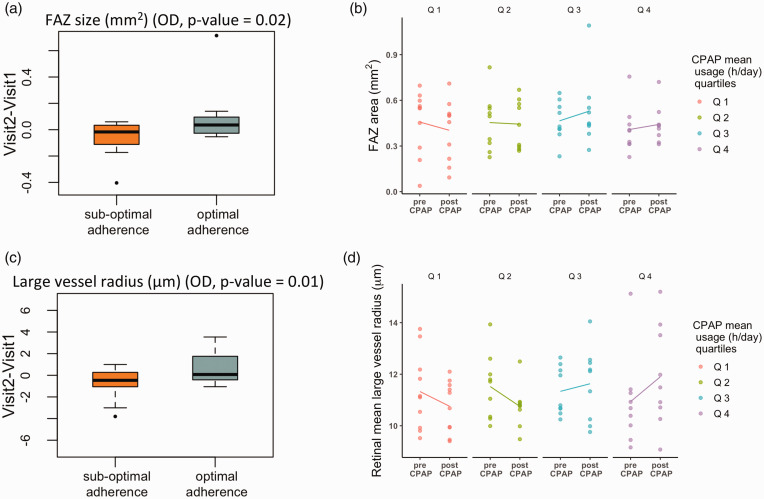
(a) Boxplots of the amount of change in FAZ area pre- and post-treatment in the right eye (OD) between participants with optimal and sub-optimal CPAP adherence. (b) FAZ area value pre- and post- treatment according to CPAP average usage (hr/day) and (c) Boxplots of the amount of change in large vessel radius. (d) Radius value in large vessels pre- and post- treatment. Quartile (Q) Q1: lowest CPAP mean usage; Q4: greatest CPAP mean usage.

Having established baseline associations between retinal phenotypes and sleep variables, we turn our attention to investigating how CPAP treatment modulates them. First, we analyzed the amount of change over time in retinal phenotypes, independently for each eye, between participants with optimal and sub-optimal CPAP adherence. Only changes in the right eye were statistically significant (OD), possibly due to the larger number of follow-up images available. Compared to individuals with sub-optimal adherence, participants with optimal adherence had a larger reduction in the parafoveal and foveal vessel densities (p-value = 0.03 and p-value = 0.01), and a greater increase in FAZ area (p-value = 0.02) and large vessel radius (p-value = 0.01). Figure 4 shows further exploration of the direction of change in FAZ area and large vessel radius measurements according to CPAP therapy by inspecting values pre- and post-treatment in individuals grouped in quartiles according to CPAP usage (hr/days).

Using the data from both eyes in the GEE model, and adjusting for confounding factors, higher average CPAP usage per night was associated with a reduction in vessel density in the foveal region (p-value = 0.02), and an increase in FAZ area (p-value = 0.03), large vessel radius (p-value = 0.01), and bifurcation distance in large vessels (p-value = 0.02) (Table 2).

**Table 2.** Summary table of standardized coefficients, p-values (significant in bold), and 95% CI, obtained using the GEE model corrected for age, sex, BMI, SBP, diabetes, hypercholesterolemia, and smoking status.

Retinal phenotypes	estimates	p-values	CI
Parafoveal VD	−0.21	0.08	(−0.45, 0.03)
Foveal VD	−0.20	**0.02**	(−0.38, −0.03)
Branching points (count)	−0.14	0.40	(−0.48, 0.19)
FAZ area	0.20	**0.03**	(0.02, 0.38)
Large vessels tor	−0.03	0.84	(−0.31, 0.25)
Small vessel tor	0.21	0.07	(−0.02, 0.45)
Large vessel radius	0.33	**0.01**	(0.08, 0.57)
Small vessel radius	−0.23	0.10	(−0.50, 0.05)
KS score	0.11	0.42	(−0.15, 0.37)
Branching symmetry ratio	0.11	0.30	(−0.09, 0.30)
Mean bifurcation distance (L)	0.28	**0.02**	(0.04, 0.51)
Mean bifurcation distance (S)	0.16	0.34	(−0.16, 0.47)
pRNFL thickness (G)	0.18	0.16	(−0.07, 0.42)

VD: vessel density; FAZ: foveal avascular zone; KS: Kolmogorov-Smirnov; L: large vasculature; S: small vessels; G: global thickness.

Next, we analyzed changes in the macular thickness associated with CPAP therapy. Before treatment, our results showed a thickening in the central part of the macula in individuals suffering with severe OSA compared to those classed as moderate OSA (p-value = 0.04) (Table 3). We further examined longitudinal changes in thickness between participants with optimal and sub-optimal CPAP adherence according to OSA severity. No statistically significant changes in macular thickness were observed in participants with moderate OSA. In severe OSA cases, after treatment individuals with better adherence showed an increase of overall macular volume (p-value <0.01), whereas participants with sub-optimal CPAP usage exhibited a reduction of macular volume (p-value <0.01) and thinning in the superior (p-value = 0.02) and temporal inner macular regions (p-value <0.01) (see Table 3).

**Table 3.** Mean and standard deviation of macular thickness for each region of interest in the parafoveal and foveal region in moderate and severe OSA cases; before treatement initiation, and pre- and post- CPAP treatment comparison.

		Both Eyes					
Inner macular thickness (μm)		Moderate (n = 10)		Severe (n = 32)	p-values*		
Foveal, mean (SD)		275.4 ± 12.7		286.5 ± 26.0	**0.04**		
Superior, mean (SD)		349.4 ± 15.7		348.0 ± 17.5	0.74		
Nasal, mean (SD)		350.0 ± 13.1		350.5 ± 18.3	0.89		
Inferior, mean (SD)		343.9 ± 14.5		345.1 ± 19.9	0.93		
Temporal, mean (SD)		334.4 ± 12.4		334.7 ± 18.4	0.94		
Macular Volume, mean (SD)		8.68 ± 0.5		8.74 ± 0.4	0.69		
	Optimal adherence (moderate = 3)			Sub-optimal adherence (moderate n = 7)			
	Pre-Treatment	Post-Treatment	p-value**	Pre-Treatment		Post-Treatment	p-value**
Foveal	284.3 ± 7.7	282.3 ± 5.9	0.34	273.1.±12.9		277.8 ± 12.6	0.07
Superior	362.0 ± 24.3	362.0 ± 24.3	1.00	346.7 ± 11.9		345.7 ± 11.5	0.49
Nasal	349.3 ± 23.7	348.5 ± 22.8	0.32	340.4 ± 12.8		339.8 ± 12.5	0.48
Inferior	353.3 ± 25.5	347.3 ± 25.3	0.22	341.6 ± 10.3		341.1 ± 8.2	0.78
Temporal	348.3 ± 26.6	347.5 ± 25.2	0.65	340.7 ± 11.2		341.3 ± 11.4	0.55
Macular Volume	8.98 ± 0.95	8.97 ± 0.93	0.50	8.61 ± 0.36		8.62 ± 0.29	0.57
	Optimal adherence (severe n = 16)			Sub-optimal adherence (severe n = 14)			
	Pre-Treatment	Post-Treatment	p-value**	Pre-Treatment		Post-Treatment	p-value**
Foveal	279.3 ± 23.7	279.1 ± 24.4	0.81	290.9 ± 28.3		290.7 ± 33.1	0.87
Superior	343.3 ± 18.6	344.2 ± 19.6	0.05	354.6 ± 14.5		352.5 ± 14.8	**0.02**
Nasal	337.4 ± 21.3	3379.5 ± 21.7	0.35	348.6 ± 17.5		345.9 ± 18.2	0.68
Inferior	339.5 ± 20.3	340.5 ± 21.3	0.05	351.7 ± 18.3		347.7 ± 18.4	0.09
Temporal	336.3 ± 20.6	336.8 ± 21.5	0.43	349.6 ± 17.5		345.7 ± 18.6	**<0.01**
Macular Volume	8.65 ± 0.45	8.69 ± 0.45	**<0.01**	8.90 ± 0.32		8.85 ± 0.29	**<0.01**

* *t-test* for variables with a normal distribution and the *Mann-Whitney U* test for non-normally distributed numerical variables. **Paired *t-test*.

## Discussion

OSA is a common sleep disorder that affects millions of people globally. If left untreated, OSA can lead to serious health complications, including high blood pressure, heart disease, and type 2 diabetes.^52–55^ SVD is also a common disorder that increases with age, vascular risk factors, is a very common cause of stroke and cognitive impairment, and is also associated with OSA.^3^

In this study, we take advantage of advanced retinal imaging modalities, OCT and OCT-A, to investigate macular thickness and vascular changes in connection to moderate/severe OSA. As individuals with OSA are at higher risk of developing SVD,^3^ we carried out an exploratory analysis of associations between retinal phenotypes and neuroimaging markers of SVD burden, as well as retinal measurements and OSA clinical variables. We analyzed retinal phenotypes at baseline and after at least 4 months of CPAP treatment to capture microvascular alterations due to OSA before and at the end of the study treatment period. Considering the importance of patients’ compliance for the efficacy of the treatment, we investigated longitudinal changes in the retinal measurements in relation to CPAP adherence.

Exploratory analyses between retinal phenotypes and SVD features showed that vessel morphologies, (i.e., vessel radius and tortuosity) could be associated with WMH severity (Fazekas scale) and PVS-CSO metrics, even in this population with sparse WMH. Retinal phenotypes characterizing the foveal region (e.g., foveal vessel density and FAZ area) were mainly associated with PVS-BG measurements. Additionally, thinning of the pRNFL layer was associated with brain atrophy score. As the pRNFL plays a crucial role in transmitting visual information from the retina to the brain through the optic nerve, a reduction in thickness of this layer suggests damage and loss of nerve fibers due to the inflammatory process and can be reflective of damage or loss of nerve cells in the brain.

Further cross-sectional analyses at baseline showed smaller vessel radius was associated with OSA severity. This result is in line with previously reported vessel narrowing in fundus imaging and might be associated with the hypoxia status of the retina due to the intermittent breathing pattern and corresponding decreases in oxygen saturation.^13–15^ Branching symmetry ratio, a measurement related to branches caliber at their bifurcation points, were also smaller in those with most severe OSA. These findings may reflect vascular remodeling resulting in hemodynamic changes as the new distribution of vessel sizes will impact retinal blood flow and oxygen transport.

After 4 months of CPAP treatment, we observed a decrease in vessel density, and an increase in FAZ area and large vessel radius between patients with optimal and sub-optimal CPAP adherence. After adjusting the model for key covariates, better CPAP usage (average per day) was still associated with increased FAZ area, large vessel radius, and greater reduction in foveal vessel density from baseline. Alterations in vessel radius observed in connection to CPAP usage might be an indication of the efficacy of the treatment reversing the vessel narrowing associated with disease severity, whereas the increase in FAZ area, bifurcation distance and the decrease in foveal vessel density might be the results of changes in macular thickness. Previous studies have reported swelling in the central macula in relation to OSA severity which may cause the displacement and/or remodeling of the vasculature across layers.^26^ Our OCT thickness analysis confirmed the thicker macula in the foveal region in participants with severe OSA. This might be triggered by the high need of oxygen of the visual cells focused on the foveal region, lack of which can cause oxidative stress and inflammation, and might lead to swelling.^56^ After CPAP treatment, patients with optimal adherence showed an overall increase in macular volume, possibly due to the thickening of the inner macular regions surrounding the fovea, whereas participants with sub-optimal adherence showed a decrease in macular volume due to thinning of the superior and temporal macular thickness.

Strengths of this study include the investigation of novel structural and functional microvascular measurements, the carefully blinded analysis, the first application of OCT-A to monitor microvascular changes and treatment effectiveness in sleep apnoea, and the exploratory analysis of associations between retinal microvasculature and neuroimaging markers of SVD in individuals with OSA.

Limitations of this study include that CPAP treatment was not randomized (it was considered unethical to withhold CPAP from patients with OSA), the lack of a control group to compare the retinal phenotypes with healthy individuals (although this would be confounded by other differences such as lack of vascular risk factors and medications all of which affect vascular function), the small sample size, and the generalization of measurements to the entire vasculature without the distinction between arteries and veins. Despite being ordinal variables, brain measurements with few or no observations in the higher categories, like the Fazekas scale and brain atrophy scores, were used as continuous to avoid overfitting the model. HSAT using PAT technology was utilized instead of the reference-standard measure of sleep, PSG. Whilst it could be argued that use of PSG would be optimal, meta-analysis has shown that respiratory indices measured by WatchPAT correlate well with PSG in patients with OSA.^57^ Furthermore, HSAT using PAT is recommended clinically by published international guidelines for the diagnosis of moderate/severe OSA in patients with a high pre-test probability^58^; the study participants here had already been diagnosed clinically using PSG or traditional HSAT prior to baseline PAT testing for the purposes of the study.

Further research should be undertaken to elucidate the underlying mechanisms that influence both retinal and cerebral microvasculature in individuals with OSA. This will help determine the extent to which the retina can serve as a reliable surrogate for cerebrovascular health. Addressing these questions has the potential to open doors to innovative, fast, and non-invasive methods for evaluating brain health, diagnosing diseases, monitoring disease progression, and assessing the effectiveness of treatments.

In conclusion, the current study reports on novel findings that suggests the potential clinical utility of OCT-A for monitoring potential treatment for SVD, here demonstrated with CPAP’s effectiveness in OSA and provides further evidence suggesting the retinal microvasculature as a potential source for surrogate biomarkers of cerebrovascular health.

## Supplemental Material

sj-pdf-1-jcb-10.1177_0271678X241291958 - Supplemental material for Retinal microvascular phenotypes can track small vessel disease burden and CPAP treatment effectiveness in obstructive sleep apnoea Supplemental material, sj-pdf-1-jcb-10.1177_0271678X241291958 for Retinal microvascular phenotypes can track small vessel disease burden and CPAP treatment effectiveness in obstructive sleep apnoea by Ylenia Giarratano, Elizabeth A Hill, Charlene Hamid, Stewart Wiseman, Calum Gray, Francesca M Chappell, Roberto Duarte Coello, Maria C Valdés-Hernández, Lucia Ballerini, Michael S Stringer, Michael J Thrippleton, Daniela Jaime Garcia, Xiaodi Liu, William Hewins, Yajun Cheng, Sandra E Black, Andrew Lim, Rosa Sommer, Joel Ramirez, Bradley J MacIntosh, Rosalind Brown, Fergus Doubal, Tom MacGillivray, Joanna M Wardlaw, Renata Riha and Miguel O Bernabeu in Journal of Cerebral Blood Flow & Metabolism
