# Supplementary material for: Retinal microvascular phenotypes can track small vessel disease burden and CPAP treatment effectiveness in obstructive sleep apnea
Source: J Cereb Blood Flow Metab. 2024 Nov 2;45(4):690–702. doi: 10.1177/0271678X241291958 (PMC11563513; doi:10.1177/0271678X241291958)
Supplement: sj-pdf-1-jcb-10.1177_0271678X241291958 - Supplemental material for Retinal microvascular phenotypes can track small vessel disease burden and CPAP treatment effectiveness in obstructive sleep apnoea [file sj-pdf-1-jcb-10.1177_0271678X241291958.pdf]

Supplementary Materials

Retinal microvascular phenotypes can track small vessel disease burden and CPAP treatment effectiveness in obstructive sleep apnoea

Ylenia Giarratano[1], Elizabeth A Hill[2,3], Charlene Hamid[2], Stewart Wiseman[2], Calum Gray[2], Francesca M Chappell[2], Roberto Duarte Coello[2], Maria C Valdés-Hernández[2], Lucia Ballerini[2,4], Michael S Stringer[2], Michael J Thrippleton[2], Daniela Jaime Garcia[2], Xiaodi Liu[2,5], William Hewins[2], Yajun Cheng[6], Sandra E Black[7], Andrew Lim[7], Rosa Sommer[7], Joel Ramirez[7], Bradley J MacIntosh[7], Rosalind Brown[2], Fergus Doubal[2], Tom MacGillivray[2], Joanna M Wardlaw[2]*, Renata Riha[2,8]*, Miguel O Bernabeu[1,9]*

(* Equally contributing senior authors)

1. Centre for Medical Informatics, Usher Institute, The University of Edinburgh, Edinburgh, UK
2. Centre for Clinical Brain Sciences, Edinburgh Imaging, UK Dementia Research Institute Centre, The University of Edinburgh, Edinburgh, UK
3. School of Applied Sciences, University of the West of England (UWE), Bristol, UK
4. University of Foreigners of Perugia, Perugia, Italy
5. Division of Neurology, Department of Medicine, The University of Hong Kong, Hong Kong SAR, China
6. Department of Neurology, West China Hospital, Sichuan University, Chengdu, China
7. Sunnybrook Health Sciences Centre, Toronto, Canada
8. Sleep Research Unit, Department of Sleep Medicine, Royal Infirmary of Edinburgh, Edinburgh, UK
9. Bayes Centre, The University of Edinburgh, Edinburgh, UK

Table S1: Sleep apnoea study demographics and brain measurements at baseline, divided by groups according to OSA severity.

**OSA Severity**	**Moderate (n = 10)**	**Severe (33)**	**p-values**
Age, years, mean (SD)	47.6 ± 8.7	50.8 ± 9.2	0.33
Female, n (%)	4(40%)	10(30%)	0.85
Hypertension, n (%)	1(10%)	16(49%)	0.07
Hypercholesterolemia, n (%)	1(10%)	11(33%)	0.30
Diabetes, n (%)	0	4(12%)	0.59
BMI (kg/m^2), mean (SD)	32.6 ± 6.6	35.5 ± 5.9	0.23
Never-Smoker, n (%)	6(60%)	16(49%)	0.78
pAHI, events/hr, mean (SD)	20.8 ± 5.5	63.6 ± 18.7	**<0.01**
SpO_2 mean, mean (SD)	94.4 ± 1.3	92.5 ± 2.0	**<0.01**
SpO_2 nadir, mean (SD)	82.1± 8.1	73.8 ± 8.8	**0.01**
**Neuroimaging**	**Moderate (n = 10)**	**Severe (32)**	**p-values**
Intracranial volume (ml), mean (SD)	1475.77 ± 132.85	1476.13 ± 171.21	1.00
WMH vol, median (IQR)	0.99 (0.84-1.51)	1.01 (0.51-1.82)	0.49
Fazekas scale (0:6), median (IQR)	2 (2-2)	1 (1-2)	**0.02**
Brain vol (ml), mean (SD)	1146.84 ± 114.62	1138.28 ± 120.94	0.84
Superficial atrophy (0:6), median (IQR)	1 (1-1)	1 (1-2)	0.36
Deep atrophy (0:6), median (IQR)	1 (1-1)	1 (1-3)	0.09
PVS vol in BG (%ROIV), median (IQR)	1.90 (1.60-2.30)	1.91 (1.62-2.61)	0.76
PVS vol in CSO (%ROIV), median (IQR)	2.99 (2.16-3.63)	3.06 (2.13-4.52)	0.61
PVS count in BG, median (IQR)	114 (95-159)	125 (108-166)	0.35
PVS count in CSO, median (IQR)	586 (419-651)	608 (477-752)	0.54
PVS size in BG (ml), median (IQR)	0.02 (0.01-0.02)	0.02 (0.01-0.02)	0.99
PVS size in CSO (ml), median (IQR)	0.02 (0.01-0.02)	0.02 (0.01-0.02)	0.78
PVS length in BG (voxels), mean (SD)	6.47 ± 0.41	6.35 ± 0.46	0.43
PVS length in CSO (voxels), mean (SD)	6.26 ± 0.37	6.43 ± 0.62	0.27
PVS width in BG (voxels), mean (SD)	3.04 ± 0.18	3.02 ± 0.29	0.82
PVS width in CSO (voxels), mean (SD)	5.67 ± 0.58	5.72 ± 0.64	0.82

WMH: white matter hyperintensity, PVS: perivascular spaces, BG: basal ganglia, CSO: centrum semiovale. IQR: interquartile range. %ROIV: as percentage of volume region of interest. Non-normal distributions are reported as medians (interquartile range, Q1–Q3).

P-values were computed using the *t-test* for variables with a normal distribution and the *Mann-Whitney U test* for non-normally distributed numerical variables. *Chi-squared test* was used for categorical variables.

Table S2: OCT-A and OCT retinal measurements at baseline and follow-up visit.

	Baseline					
	Right eye			Left eye		
**OCT-A**	**Moderate (n = 9)**	**Severe (n = 31)**	**Total (n = 40)**	**Moderate (n = 8)**	**Severe (n = 29)**	**Total (n = 37)**
Parafoveal VD, mean (SD)	0.32 ± 0.04	0.3 ± 0.04	0.31 ± 0.04	0.26 ± 0.05	0.27 ± 0.04	0.27 ± 0.04
Foveal VD, mean (SD)	0.08 ± 0.05	0.08 ± 0.04	0.08 ± 0.04	0.05 ± 0.03	0.07 ± 0.04	0.07 ± 0.04
Branching points count, median (IQR)	751 (640-954)	740 (588-829)	740 (603-842)	654 (531-793)	622 (498-716)	634 (510-742)
FAZ area (mm^2), mean (SD)	0.53 ± 0.17	0.42 ± 0.16	0.44 ± 0.17	0.57 ± 0.15	0.43 ± 0.17	0.46 ± 0.18
Large vessel tortuosity, mean (SD)	1.15 ± 0.15	1.1 ± 0.01	1.11 ± 0.07	1.1 ± 0.01	1.1 ± 0.02	1.1 ± 0.02
Small vessel tortuosity, mean (SD)	1.14 ± 0.01	1.14 ± 0.01	1.14 ± 0.01	1.14 ± 0.01	1.14 ± 0.01	1.14 ± 0.01
Large vessel radius (µm), mean (SD)	11.98 ± 1.28	11.06 ± 1.25	11.27 ± 1.3	12.33 ± 2.07	11.18 ± 1.4	11.41 ± 1.58
Small vessel radius (µm), mean (SD)	5.77 ± 0.39	5.78 ± 0.39	5.78 ± 0.39	5.82 ± 0.32	5.72 ± 0.42	5.74 ± 0.39
KS score, mean (SD)	0.16 ± 0.02	0.18 ± 0.02	0.18 ± 0.02	0.18 ± 0.02	0.18 ± 0.02	0.18 ± 0.02
Branching asymmetry ratio, mean (SD)	0.90 ± 0.02	0.90 ± 0.02	0.90 ± 0.02	0.91 ± 0.01	0.90 ± 0.01	0.90 ± 0.01
Large vessel bifurcation distance (µm), mean (SD)	260.77 ± 40.55	264.36 ± 40.49	263.55 ± 40.01	257.30 ± 47.77	263.73 ± 46.12	262.34 ± 45.88
Small vessel bifurcation distance (µm), mean (SD)	71.96 ± 6.54	72.5 ± 8.11	72.39 ± 7.73	71.83 ± 8.5	71.74 ± 6.93	71.76 ± 7.17
**OCT**	**Moderate (n = 10)**	**Severe (n = 28)**	**Total (n = 38)**	**Moderate (n = 10)**	**Severe (n = 29)**	**Total (n = 39)**
pRNFL thickness (µm), mean (SD)	99.60 ± 10.07	94.71 ± 10.59	96.00 ± 10.60	96.00 ± 9.99	95.70 ± 10.60	95.80 ± 10.32
	**Follow-up (post treatment)**					
	**Right eye**			**Left eye**		
**OCT-A**	**Moderate (n = 9)**	**Severe (n = 28)**	**Total (n = 37)**	**Moderate (n = 8)**	**Severe (n = 23)**	**Total (n = 31)**
Parafoveal VD, mean (SD)	0.32 ± 0.03	0.31 ± 0.03	0.31 ± 0.03	0.28 ± 0.04	0.28 ± 0.03	0.28 ± 0.03
Foveal VD, mean (SD)	0.07 ± 0.03	0.08 ± 0.04	0.08 ± 0.04	0.06 ± 0.03	0.07 ± 0.03	0.06 ± 0.03
Branching points count, median (IQR)	767 ± 211	751 ± 157	756 ± 171	647 ± 254	648 ± 192	647 ± 206
FAZ area (mm^2), mean (SD)	0.51 ± 0.13	0.44 ± 0.2	0.45 ± 0.18	0.49 ± 0.11	0.44 ± 0.18	0.46 ± 0.16
Large vessel tortuosity, mean (SD)	1.10 ± 0.05	1.10 ± 0.02	1.10 ± 0.03	1.10 ± 0.01	1.10 ± 0.02	1.10 ± 0.02
Small vessel tortuosity, mean (SD)	1.14 ± 0.01	1.14 ± 0.01	1.14 ± 0.01	1.14 ± 0.01	1.14 ± 0.01	1.14 ± 0.01
Large vessel radius (µm), mean (SD)	11.28 ± 0.82	11.23 ± 1.56	11.24 ± 1.4	11.89 ± 1.7	11.15 ± 0.98	11.33 ± 1.2

Small vessel radius (µm), mean (SD)	5.92 ± 0.15	5.8 ± 0.48	5.83 ± 0.42	5.9 ± 0.31	6.16 ± 1.39	6.09 ± 1.18
KS score, mean (SD)	0.17 ± 0.02	0.17 ± 0.02	0.17 ± 0.02	0.18 ± 0.02	0.17 ± 0.02	0.17 ± 0.02
Branching asymmetry ratio, mean (SD)	0.90 ± 0.01	0.90 ± 0.01	0.90 ± 0.01	0.90 ± 0.01	0.90 ± 0.02	0.90 ± 0.01
Large vessel bifurcation distance (µm), mean (SD)	245.53 ± 26.16	266.90 ± 44.96	261.70 ± 41.89	262.52 ± 41.70	270.9 ± 55.30	268.67 ± 51.47
Small vessel bifurcation distance (µm), mean (SD)	72.27 ± 7.38	72.66 ± 6.27	72.56 ± 6.46	73.18 ± 9.1	72.17 ± 5.55	72.45 ± 6.55
**OCT**	**Moderate (n = 10)**	**Severe (n = 26)**	**Total (n = 36)**	**Moderate (n = 10)**	**Severe (n = 28)**	**Total (n = 38)**
pRNFL thickness (µm), mean (SD)	99.60 ± 10.07	94.81 ± 11.00	96.14 ± 10.82	96.00 ± 9.99	95.86 ± 10.78	95.90 ± 10.44

VD: vessel density, FAZ: Foveal avascular zone, KS: Kolmogorov-Smirnov.

Table S3: Summary tables of estimates, p-values, and 95% CI, obtained using the GEE model corrected for age, sex, BMI, SBP, diabetes, hypercholesterolemia, smoking status, and OCT-A image quality.

Fazekas scale

Retinal_phenotypes	*estimates*	*p-values*	*lower*	*upper*
Parafoveal VD	-0.02	0.68	-0.13	0.09
Foveal VD	0.06	0.29	-0.05	0.18
Branching points (count)	0.00	1	-0.12	0.12
FAZ area	-0.03	0.65	-0.17	0.11
Large vessels tor	0.03	0.16	-0.01	0.08
Small vessel tor	-0.01	0.85	-0.11	0.09
Large vessel radius	0.12	**0.01**	0.03	0.22
Small vessel radius	0.14	**0.04**	0.01	0.27
KS score	0.01	0.74	-0.07	0.10
Symmetry ratio	0.12	**0.01**	0.03	0.20
Bifurcation distance (L)	-0.02	0.48	-0.08	0.04
Bifurcation distance (S)	0.00	0.95	-0.14	0.15
RNFL thickness (G)	-0.01	0.84	-0.15	0.12

Atrophy Deep

Retinal_phenotypes	*estimates*	*p-values*	*lower*	*upper*
Parafoveal VD	-0.06	0.16	-0.15	0.02
Foveal VD	-0.02	0.70	-0.13	0.09
Branching points (count)	-0.09	0.14	-0.20	0.03
FAZ area	0.01	0.87	-0.10	0.11
Large vessels tor	-0.04	0.50	-0.17	0.08
Small vessel tor	0.01	0.77	-0.07	0.10
Large vessel radius	-0.01	0.81	-0.11	0.09
Small vessel radius	-0.02	0.69	-0.14	0.10
KS score	0.11	**0.03**	0.01	0.21
Symmetry ratio	0.03	0.66	-0.09	0.14
Bifurcation distance (L)	0.01	0.89	-0.07	0.08
Bifurcation distance (S)	0.08	0.245	-0.06	0.22
RNFL thickness (G)	-0.20	**<0.01**	-0.32	-0.08

PVS BG total volume

Retinal_phenotypes	*estimates*	*p-values*	*lower*	*upper*
Parafoveal VD	-0.06	0.736	-0.39	0.28
Foveal VD	0.43	**<0.01**	0.18	0.69
Branching points (count)	-0.07	0.399	-0.23	0.09
FAZ area	-0.38	**0.01**	-0.67	-0.10
Large vessels tor	0.03	0.382	-0.04	0.11
Small vessel tor	-0.01	0.853	-0.14	0.12
Large vessel radius	0.17	0.367	-0.20	0.54
Small vessel radius	0.29	0.147	-0.10	0.67
KS score	0.09	0.267	-0.07	0.24
Symmetry ratio	0.22	**0.04**	0.01	0.44
Bifurcation distance (L)	-0.06	0.629	-0.28	0.17
Bifurcation distance (S)	0.02	0.877	-0.22	0.25
RNFL thickness (G)	0.08	0.58	-0.19	0.35

PVS BS count

Retinal_phenotypes	*estimates*	*p-values*	*lower*	*upper*
Parafoveal VD	0.01	0.82	-0.07	0.09
Foveal VD	0.09	**<0.01**	0.05	0.13
Branching points (count)	-0.03	0.33	-0.10	0.03
FAZ area	-0.09	**<0.01**	-0.13	-0.04
Large vessels tor	0.03	0.06	-0.00	0.06
Small vessel tor	0.01	0.65	-0.04	0.07
Large vessel radius	0.06	**0.04**	0.00	0.12
Small vessel radius	0.01	0.8	-0.08	0.10
KS score	0.04	0.22	-0.02	0.09
Symmetry ratio	0.03	0.36	-0.04	0.10
Bifurcation distance (L)	0.04	0.08	-0.00	0.08
Bifurcation distance (S)	0.03	0.41	-0.05	0.12
RNFL thickness (G)	0.06	0.31	-0.06	0.17

PVS CSO total volume				
Retinal_phenotypes	*estimates*	*p-values*	*lower*	*upper*
Parafoveal VD	-0.08	0.53	-0.33	0.17
Foveal VD	0.27	**0.03**	0.03	0.51
Branching points (count)	-0.07	0.48	-0.25	0.12
FAZ area	-0.25	0.06	-0.51	0.01
Large vessels tor	0.05	0.27	-0.04	0.15
Small vessel tor	-0.07	0.28	-0.19	0.05
Large vessel radius	0.22	0.16	-0.09	0.53
Small vessel radius	0.30	0.07	-0.03	0.63
KS score	0.06	0.42	-0.08	0.20
Symmetry ratio	0.22	**0.02**	0.03	0.40
Bifurcation distance (L)	-0.09	0.37	-0.28	0.10
Bifurcation distance (S)	-0.01	0.9	-0.25	0.22
RNFL thickness (G)	0.14	0.32	-0.14	0.42

PVS CSO count				
Retinal_phenotypes	*estimates*	*p-values*	*lower*	*upper*
Parafoveal VD	-0.04	0.27	-0.11	0.03
Foveal VD	0.07	0.07	-0.01	0.14
Branching points (count)	-0.03	0.35	-0.11	0.04
FAZ area	-0.09	0.05	-0.18	-0.00
Large vessels tor	0.06	**0.01**	0.02	0.11
Small vessel tor	0.00	0.98	-0.04	0.05
Large vessel radius	0.01	0.76	-0.06	0.08
Small vessel radius	-0.08	**0.02**	-0.14	-0.01
KS score	0.02	0.64	-0.05	0.09
Symmetry ratio	-0.01	0.8	-0.07	0.06
Bifurcation distance (L)	0.03	0.11	-0.01	0.07
Bifurcation distance (S)	0.02	0.53	-0.05	0.10
RNFL thickness (G)	0.05	0.34	-0.06	0.17

VD: vessel density, FAZ: Foveal avascular zone, KS: Kolmogorov-Smirnov, L: large vasculature, S: small vessels, G: global thickness. PVS: perivascular spaces, BG: basal ganglia, CSO: centrum semiovale.

Table S4: Summary tables of estimates, p-values, and 95% CI, obtained using the GEE model corrected for age, sex, BMI, SBP, diabetes, hypercholesterolemia, smoking status, and OCT-A image quality.

pAHI index				
Retinal_phenotypes	*estimates*	*p-values*	*lower*	*upper*
Parafoveal VD	0.16	0.10	-0.03	0.35
Foveal VD	0.09	0.58	-0.23	0.40
Branching points (count)	0.05	0.63	-0.15	0.25
FAZ area	-0.16	0.27	-0.45	0.12
Large vessels tor	-0.06	0.20	-0.15	0.03
Small vessel tor	0.06	0.59	-0.16	0.27
Large vessel radius	-0.21	**0.05**	-0.42	0.00
Small vessel radius	-0.15	0.30	-0.43	0.13
KS score	-0.10	0.38	-0.31	0.12
Branching symmetry ratio	-0.24	**<0.01**	-0.41	-0.07
Bifurcation distance (L)	0.07	0.42	-0.10	0.24
Bifurcation distance (S)	-0.10	0.35	-0.31	0.11
pRNFL thickness (G)	0.16	0.18	-0.07	0.38

Mean SpO_2				
Retinal_phenotypes	*estimates*	*p-values*	*lower*	*upper*
Parafoveal VD	-0.06	0.48	-0.24	0.11
Foveal VD	-0.05	0.62	-0.27	0.16
Branching points (count)	-0.01	0.90	-0.22	0.20
FAZ area	0.09	0.41	-0.13	0.32
Large vessels tor	0.02	0.76	-0.09	0.12
Small vessel tor	-0.01	0.94	-0.17	0.16
Large vessel radius	0.01	0.94	-0.17	0.18
Small vessel radius	-0.01	0.92	-0.25	0.23
KS score	0.10	0.28	-0.08	0.29
Branching symmetry ratio	0.05	0.52	-0.11	0.21
Bifurcation distance (L)	-0.10	0.12	-0.24	0.03
Bifurcation distance (S)	0.03	0.77	-0.16	0.22
pRNFL thickness (G)	-0.24	**0.03**	-0.45	-0.02

SpO_2 nadir				
Retinal_phenotypes	*estimates*	*p-values*	*lower*	*upper*
Parafoveal VD	-0.14	0.25	-0.38	0.10
Foveal VD	0.00	0.99	-0.25	0.25
Branching points (count)	-0.14	0.345	-0.42	0.15
FAZ area	0.10	0.41	-0.14	0.34
Large vessels tor	0.13	0.07	-0.01	0.28
Small vessel tor	0.14	0.19	-0.07	0.36
Large vessel radius	0.20	**0.01**	0.06	0.35
Small vessel radius	-0.12	0.31	-0.36	0.11
KS score	-0.09	0.33	-0.28	0.09
Branching symmetry ratio	0.22	**0.03**	0.03	0.42
Bifurcation distance (L)	-0.05	0.54	-0.19	0.10
Bifurcation distance (S)	0.21	0.16	-0.08	0.50
pRNFL thickness (G)	-0.29	**0.01**	-0.51	-0.07

VD: vessel density, FAZ: Foveal avascular zone, KS: Kolmogorov-Smirnov, L: large vasculature, S: small vessels, G: global thickness.

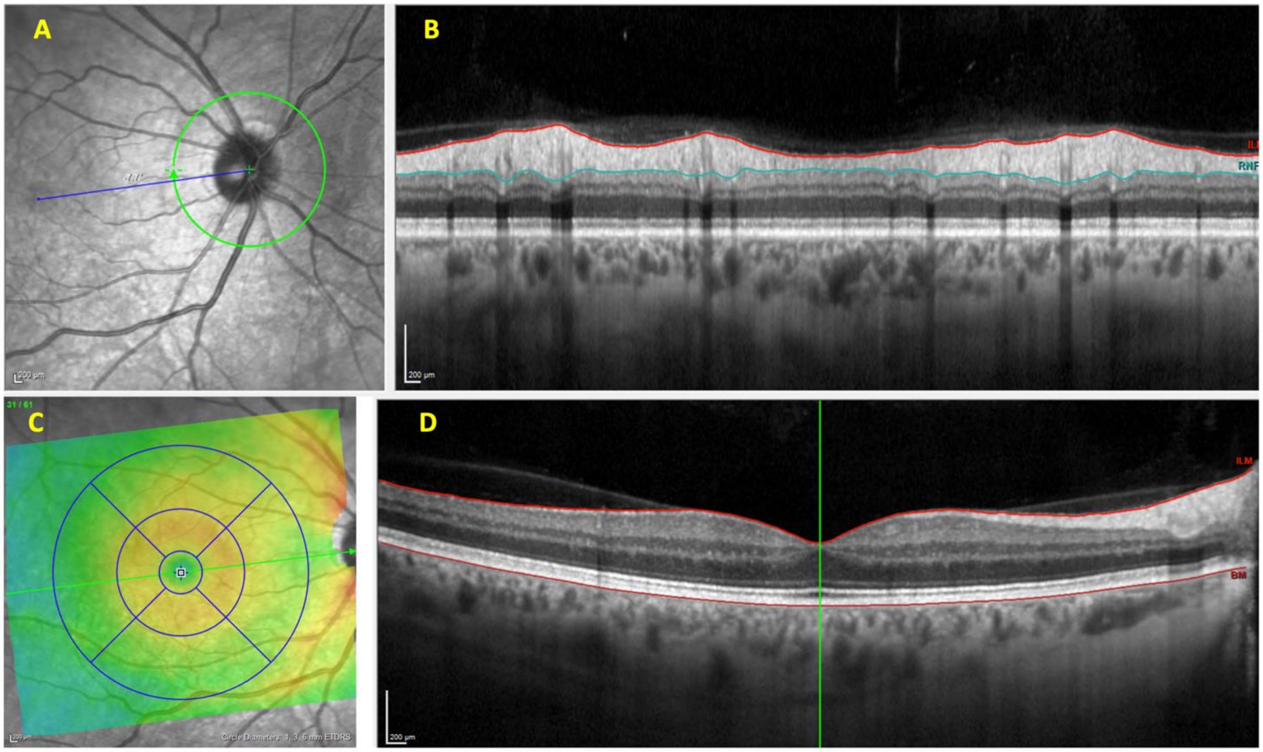

Supplementary Figure S1: (A) Acquisition of peripapillary retinal nerve fiber layer (pRNFL). (B) pRNFL segmentation. (C) Gradient representing retinal thickness in the macula with overlapping regions of interest. (D) Segmentation of the retinal layers included in the macular thickness analysis.

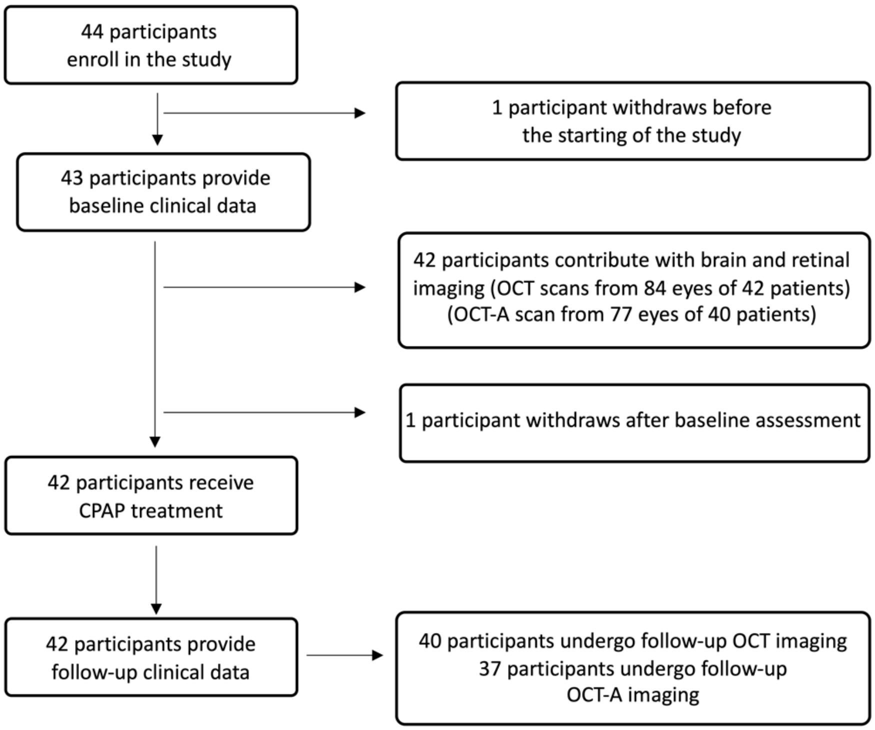

Supplementary Figure S2: Flow chart of the study process and participants.

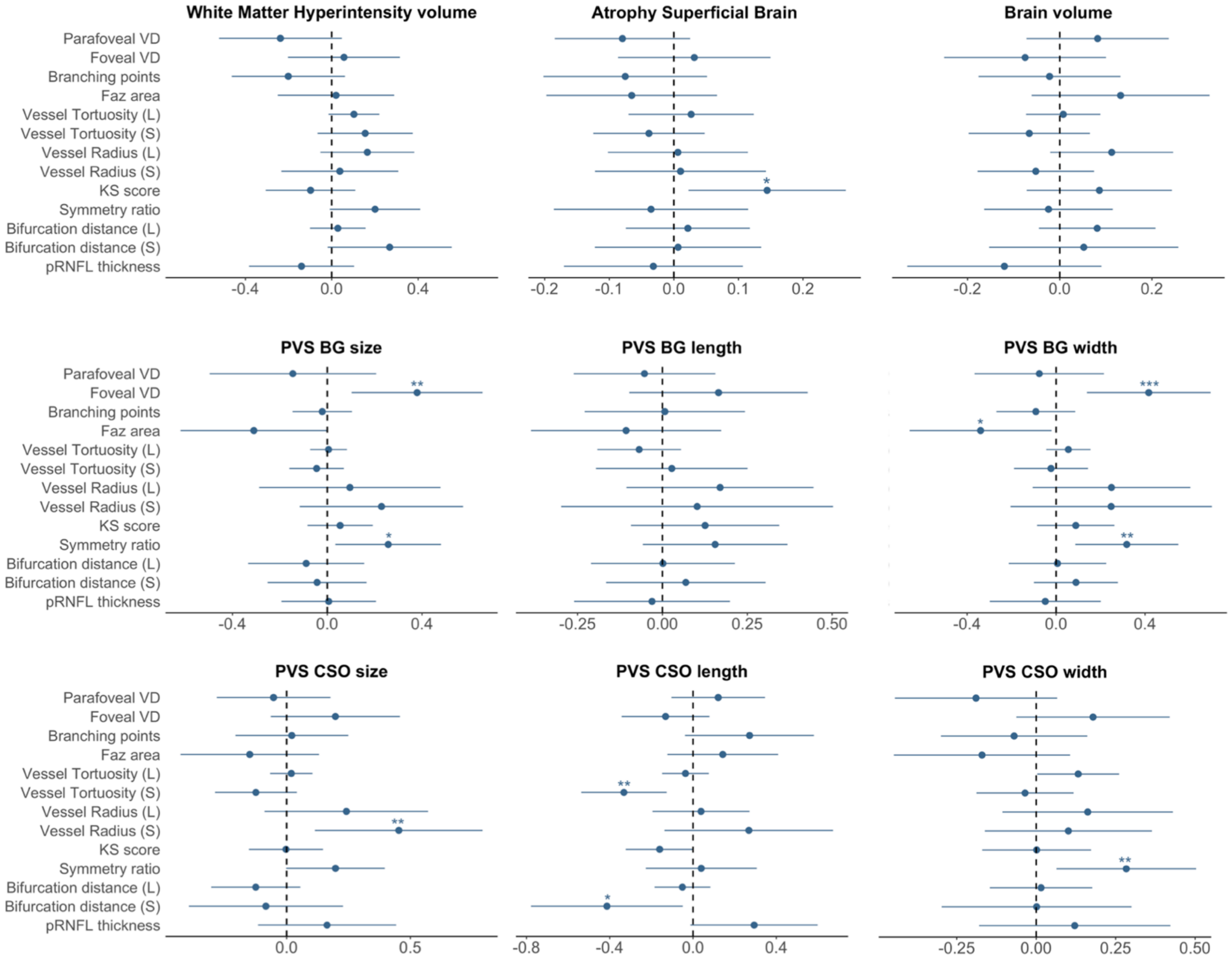

Supplementary Figure S3: Standardized betas and 95% confidence intervals obtained using a generalized estimating equations model adjusted for age, sex, systolic blood pressure, body mass index, diabetes status, hypercholesterolemia, smoking status, and OCT-A image quality index. PVS volumes were corrected for the volume of the relevant region of interest. BMI: body mass index, PVS: perivascular spaces, CSO: centrum semiovale, BG: basal ganglia. CI containing the value zero (dashed line) indicates a p-value greater than 0.05. *: p-value ≤ 0.05, **: p-value ≤ 0.01, ***: p-value ≤ 0.001. VD: vessel density, FAZ: foveal avascular zone, KS: Kolmogorov-Smirnov, L: large vasculature, S: small vessels.
